# Immunomodulatory effects of intraoperative dexmedetomidine on T helper 1, T helper 2, T helper 17 and regulatory T cells cytokine levels and their balance: a prospective, randomised, double-blind, dose-response clinical study

**DOI:** 10.1186/s12871-018-0625-2

**Published:** 2018-11-08

**Authors:** Jae-Myeong Lee, Hyo-Jo Han, Won-Kyu Choi, Subin Yoo, Soojin Baek, Jaemin Lee

**Affiliations:** 1 Department of Anesthesiology and Pain Medicine, Uijeongbu St. Mary’s Hospital, College of Medicine, The Catholic University of Korea, 271 Choenbo-ro, Uijeongbu-si, Gyeonggi-do 11765 Republic of Korea; 2 Department of Anesthesiology and Pain Medicine, CHA Bundang Medical Center, CHA University, Seongnam, South Korea

**Keywords:** Cytokines, Dexmedetomidine, Surgery, T cell, Th1/Th2 balance, T17/Treg balance

## Abstract

**Background:**

The ratio of T helper 1 (Th1) to T helper 2 (Th2) as well as T helper 17 (Th17) to regulatory T cells (Treg) represents the state and direction of immune response. Recent studies demonstrated that dexmedetomidine reduced the secretion of inflammatory cytokines. We performed this study to investigate the effect of different doses of intraoperative dexmedetomidine on the expression of Th1, Th2, T17 and Treg cytokines and their ratios.

**Methods:**

Seventy-five patients undergoing laparoscopic cholecystectomy were randomly separated into one of three groups: the full dose group (*n* = 25), in which dexmedetomidine was infused with a 1.0 μg/kg loading followed by an infusion of 0.5 μg/kg/min after anaesthetic induction, or the half dose group (*n* = 26), in which the dose was half of that of full dose group, or the saline group (*n* = 24) which was control. T cell cytokines were quantified by sandwich enzyme-linked immunoassay for blood samples taken after anaesthetic induction (T0), at the end of surgery (T1), and 60 min after surgery (T2). IFN-gamma/IL-4 and IL-17/IL-10, which represent the ratio of Th1/Th2 and Th17/Treg cytokines, respectively, were calculated as indices of immune cell levels based upon serum cytokines levels in place of direct measurements. C-reactive protein (CRP) concentrations were measured on the next day following surgery.

**Results:**

The full dose group was associated with higher ratios of IFN-gamma/IL-4 than those of half dose group and control [10.1 vs. 1.9 at T1 (*P* = 0.041) compared with half dose group, and 10.1 vs. 0.2 at T1 (*P* = 0.031), 7.4 vs. 0.1 at T2 (*P* = 0.025) compared with control]. IL-17/IL-10 ratios were higher in the full dose group than those in control [4.2 vs. 0.6 at T1 (*P* = 0.013), 3.0 vs. 0.3 at T2 (*P* = 0.011)]. The CRP levels were lower in the dexmedetomidine-treated groups in a dose-dependent manner.

**Conclusions:**

Dexmedetomidine exhibits immunomodulatory effects, shifting the Th1/Th2 and T17/Treg cytokine balance toward Th1 and T17, respectively, in a dose-dependent pattern in patients with surgical and anaesthetic stress.

**Trial registration:**

Clinical Research Information Service, Republic of Korea (CRIS); KCT0000503; Registration date: Aug 13, 2012.

## Background

Surgery itself is a significant stressor for patients receiving operation and may cause suppressed immunity. The immune-compromised response in these patients is the result of the activation of sympathetic nervous system and hypothalamic-pituitary-adrenal axis [1, 2]. Additionally, anaesthetic agents themselves have immunomodulatory activity [3].

Dexmedetomidine, an alpha-2 adrenoceptor agonist, is a useful agent for sedation and analgesia for patients admitted in intensive care units. Studies in animals and clinical trials have shown that dexmedetomidine reduces the secretion of proinflammatory cytokines, which in turn alleviates systemic inflammatory response and even reduces mortality [4, 5]. These results suggest that harmful inflammatory responses can be modulated by administering an alpha-2 agonist to patients who are stressed and have an enhanced inflammatory reaction due to surgery and anaesthesia. However, the effects of different doses of dexmedetomidine on immunomodulation during perioperative period have not been examined.

The subtype of T helper cell is determined by the differentiation of precursor helper T cells (Th0) into T helper 1 (Th1) or T helper 2 (Th2) cells. The Th1 cells produce interferon-gamma (IFN-gamma) and favor cell-mediated immune response. The Th2 cells produce IL-4 and/or IL-10, a regulatory cytokine, and favor humoral immunity by antibody production [6]. Surgery decreases the Th1/Th2 ratio, which is responsible for the suppressed cell-mediated immunity after surgery [7]. It has been recently reported that there is an IL-23-dependent Th17 cell population which produces IL-17 but not IFN-gamma or IL-4 [8]. The ratios of Th17 to regulatory T cells (Treg) as well as Th1 to Th2 represent the state and direction of immune response and play immunomodulatory role [7, 9, 10].

The purpose of this study was to analyse the effect of different doses of dexmedetomidine on the levels of Th1, Th2, Th17 and Treg cytokines and their ratios in patients under surgical and anaesthetic stress. We hypothesised that agonising alpha-2 adrenoceptors would modulate the profiles of T cytokines in a dose-dependent manner.

## Methods

Adult patients with ASA physical status class I or II who were diagnosed for chronic cholecystitis or gall bladder polyp or biliary colic and underwent laparoscopic cholecystectomy between January and December 2011 were included in this randomised, controlled, dose-response study. We excluded the patients with diagnosis of acute cholecystitis or gall bladder empyema, preoperative fever (≥ 38.3 °C) or leukocytosis (≥ 10,000/mm^3^), having preexisting illness such as diabetes mellitus and systemic inflammatory disease. The Institutional Review Board of our hospital approved the study (approval number, KC10MISI0725), and each patient gave verbal and written informed consent in advance. This study was registered with Clinical Research Information Service, Republic of Korea (CRIS) (Registration number; KCT0000503, Registration date; Aug 13, 2012).

Under standard monitoring, anaesthesia was induced with lidocaine 40 mg, propofol 2 mg/kg and rocuronium 1 mg/kg after denitrogenation with 100% oxygen. Following tracheal intubation, controlled ventilation was conducted with a tidal volume of 8 ml/kg and respiratory rate of 12–15 to maintain end-tidal CO_2_ at 30–35 mmHg. General anaesthesia was maintained with sevoflurane and continuous infusion of remifentanil.

Patients were randomly allocated into one of the following three groups using a computer generated sequence of random numbers; the full dose group (group FD, *n* = 25), the half dose group (group HD, *n* = 26), and the saline group (group S, *n* = 24). Immediately after the induction of anaesthesia, patients in the group FD were infused with a bolus dose of 1.0 μg/kg dexmedetomidine for 10 min, followed by continuous infusion at a rate of 0.5 μg/kg/min. Patients in the group HD received a loading dose of 0.5 μg/kg dexmedetomidine, followed by constant infusion of 0.25 μg/kg/min. Patients in the group S were infused with an equal volume of normal saline. The group sequence was concealed in sealed opaque envelopes which were opened just before induction of anaesthesia. Another anaesthesiologist not involved in the anaesthetic management of the patients prepared covered syringe pumps for the dexmedetomidine and saline solutions and held the randomisation codes until data analysis was complete. The patients and anaesthesiologists in charge were blinded to the group allocation.

In this study, levels of serum cytokines were measured as indices of immune cell levels in place of direct measurements by phenotyping by flow cytometry. Blood samples for cytokine analysis were collected from the external jugular vein after induction of anaesthesia (T0; the baseline value before surgery), at the end of surgery (T1), and in the post-anaesthetic care unit 1 h after surgery (T2). All blood samples were collected in standard tubes containing anticoagulant, and centrifuged at 1500 rpm for 10 min at 4 °C. Plasma was collected by pipetting and stored at − 70 °C.

Analyses of cytokine levels were performed after all samples had been collected. IFN-gamma, IL-4, IL-17 and IL-10 were quantified as the cytokines for Th1, Th2, Th-17 and Treg, respectively, by conventional enzyme-linked immunosorbent assay (ELISA) using quantitative sandwich enzyme immunoassay test kits. The minimum detectable levels of IFN-gamma, IL-4, IL-17 and IL-10 were 0.01, 0.05, 0.01 and 0.05 pg/ml, respectively. Each blood sample was analysed twice and the average value was used for interpretation. Ratios of IFN-gamma to IL-4, and IL-17 to IL-10 were calculated.

On the next day after surgery, the C-reactive protein (CRP) levels were measured to evaluate the severity of inflammatory reactions according to different doses of intraoperative dexmedetomidine. Nephelometry (Mitsubishi Kagaku Bio-Clinical Laboratories, Tokyo, Japan) was used to measure CRP concentration [11].

Based on our pilot study, the expected difference in mean value of IFN-gamma/IL-4 at T2 between the group FD and the group S was 6.5 with SD of 7.2. Based on Δ [(μ-μ′)/δ] = 0.9 (where μ-μ′ = difference in mean and δ = SD), 24 patients per group was adequate to achieve a power of 80% with an alpha error of 0.05.

To compare the demographic data and postoperative CRP levels among groups, one-way analysis of variance (ANOVA) was used after confirming normal distribution of the data by Shapiro-Wilk test. Kruskal-Wallis test was used to compare cytokine concentrations among groups at each time point, while Friedman test was used to compare cytokine concentrations within a group. All statistical analyses were performed using the SPSS® statistical package, version 15.0 (SPSS Inc., Chicago, IL, USA) for Windows®. A *P*-value of < 0.05 was considered to be statistically significant. The primary outcome of the study was to detect the immunomodulatory effect of different doses of dexmedetomidine in terms of the levels of Th1, Th2, Th17 and Treg cytokines and their ratios.

## Results

**Fig. 1:**
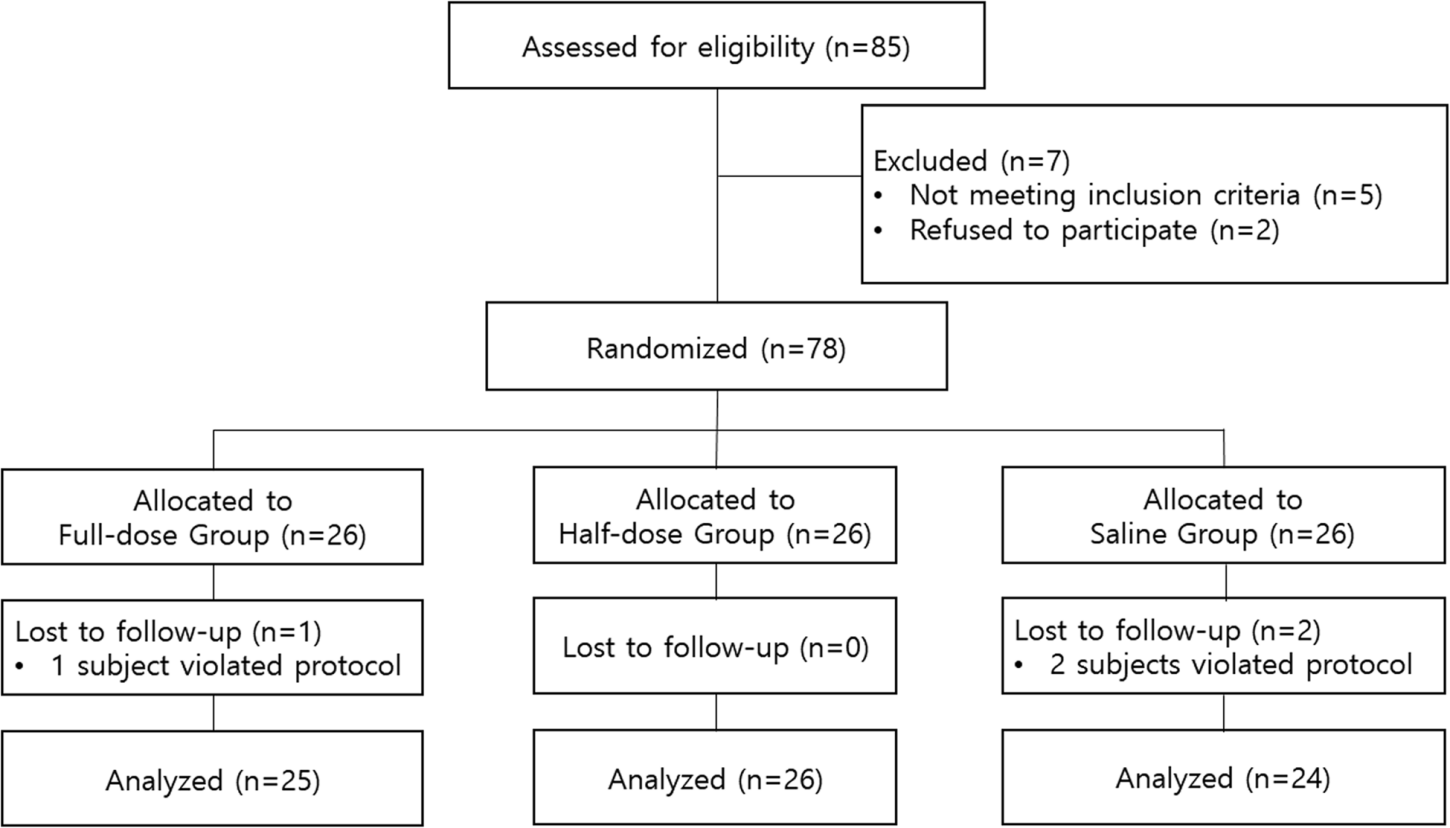
Flow diagram of randomisation and follow-up of enrolled participants

Flow diagram of randomisation is shown in Fig. 1. The characteristics of the patients are summarised in Table 1. Mean duration of surgery and anaesthesia were comparable among groups.

**Table 1:** Demographic and anaesthetic data of patients

	Group S	Group HD	Group FD
	(*n* = 24)	(*n* = 26)	(*n* = 25)
Age (yr)	41.8 ± 4.7	40.8 ± 3	40.9 ± 6.7
Height (cm)	171 ± 5.8	170.1 ± 7.5	168.1 ± 7.3
Weight (kg)	70.4 ± 6.5	66.4 ± 7.6	65.8 ± 9.2
Surgical Duration (min)	46.9 ± 20	44.9 ± 15.5	46.5 ± 14.4
Anaesthetic Duration (min)	59.9 ± 18	53.4 ± 17.1	61 ± 14.8

Values are mean ± SD

No significant difference was found among groups

*S* saline, *HD* half dose, *FD* full dose

There were no significant differences in the baseline (T0) cytokines concentrations among groups. In the group S, the level of IFN-gamma, a Th1 cytokine, significantly decreased at T1 and T2 compared to T0. However, it did not show any significant change over the period of the study in the group HD and the group FD. IFN-gamma level was significantly higher in the dexmedetomidine-treated groups than in the group S at T1 and T2. The level of IL-4, a Th2 cytokine, declined during and after the surgery in the dexmedetomidine-treated groups compared with the baseline values: the reduction in IL-4 was in dose-dependent manner. However, it did not change over the period of the study in the group S (Table 2).

**Table 2:** Time courses of IFN-gamma, IL-4 and their ratios among groups

	Group S		Group HD		Group FD	
	(*n* = 24)		(*n* = 26)		(*n* = 25)	
IFN-gamma (pg/ml)						
T0	74.2	(2.2, 141.4)	88.4	(1.6, 250.1)	87.6	(28.1, 219.3)
T1	18.8	(1.8, 48.7)*	126	(36.3, 278.7)†	203	(51.0, 386.5)†
T2	11.5	(1.3, 45.5)*	125	(10.6, 400.5)†	210	(41.6, 503.4)†
IL-4 (pg/ml)						
T0	81.6	(21.6, 145.5)	90.9	(20.7, 250.1)	91.6	(30.1, 223.4)
T1	80.7	(13.3, 209.6)	67.2	(1.4, 231.3)	20.1	(2.3, 66.7)*
T2	98.9	(26.5, 217.2)	30.1	(1.9, 99.3)	28.4	(1.2, 108.7)*
IFN-gamma/IL-4						
T0	0.9	(0.2, 2.6)	1.0	(0.2, 1.9)	1.0	(0.3, 1.3)
T1	0.2	(0.1, 0.6)*	1.9	(0.2, 6.4)†	10.1	(3.5, 15.1)*†‡
T2	0.1	(0.0, 0.3)*	4.1	(1.1, 10.4)*†	7.4	(0.8, 21.8)*†

Values are median with quartiles (25, 75%)

T0, after induction of anaesthesia (baseline); T1, at the end of surgery;

T2, 1 h after surgery at postanaesthetic care unit

*, *P* < 0.05 vs. T0; †, *P* < 0.05 vs. group S;

‡, *P* < 0.05 vs. group HD

*S* saline, *HD* half dose, *FD* full dose

IFN-gamma/IL-4 ratio showed no statistical difference at T0 among groups. In the group S, IFN-gamma/IL-4 ratio was significantly lower at T1 and T2 compared to T0, on the other hand, the ratios were higher compared to T0 in the dexmedetomidine-treated groups, with dose-dependent pattern. At T1 and T2, the dexmedetomidine-treated groups showed higher IFN-gamma/IL-4 ratios compared with those in the group S, indicating the shift of the IFN-gamma/IL-4 balance toward IFN-gamma (Table 2).

The concentration of IL-17, a T17 cytokine, increased as the surgery underwent in the dexmedetomidine-treated groups but with no statistical significance, however, no such change was evident in the group S. The level of IL-10, a regulatory cytokine, showed significant decrease during the surgery in the group FD compared with the group HD and the group S. IL-17/IL-10 ratio showed no statistical differences at T0 among the groups, but increased in the group FD compared to T0 as the surgery underwent indicating the shift of the IL-17/IL-10 balance toward IL-17 (Table 3).

**Table 3:** Time courses of IL-17, IL-10 and their ratios among groups

	Group S		Group HD		Group FD	
	(*n* = 24)		(*n* = 26)		(*n* = 25)	
IL-17 (pg/ml)						
T0	71.5	(9.8, 211.5)	77.6	(2.8, 229.2)	58.4	(21.8, 104.6)
T1	70.1	(23.4, 131.1)	190	(51.6, 326.1)	118	(8.1, 387.4)
T2	23.2	(7.1, 38.8)	116	(11.6, 280.4)	80.6	(38.1, 108.1)
IL-10 (pg/ml)						
T0	101	(10.1, 241.0)	97.6	(7.1, 210.6)	128	(21.6, 288.4)
T1	120	(24.7, 258.1)	106	(4.9, 221.7)	28.2	(1.9, 56.5)*†
T2	75.3	(1.2, 210.2)	85.4	(36.1, 228.5)	26.5	(1.3, 68.1)*
IL-17/IL-10						
T0	0.7	(0.2, 2.1)	0.8	(0.2, 2.2)	0.5	(0.1, 0.7)
T1	0.6	(0.2, 0.8)	1.8	(0.2, 5.8)	4.2	(2.1, 9.6)*†
T2	0.3	(0.1, 0.5)	1.4	(0.2, 2.7)†	3.0	(0.9, 4.5)*†

Values are median with quartiles (25, 75%)

T0, after induction of anaesthesia (baseline); T1, at the end of surgery;

T2, 1 h after surgery at postanaesthetic care unit

*, *P* < 0.05 vs. T0; †, *P* < 0.05 vs. group S

*S* saline, *HD* half dose, *FD* full dose

**Fig. 2:**
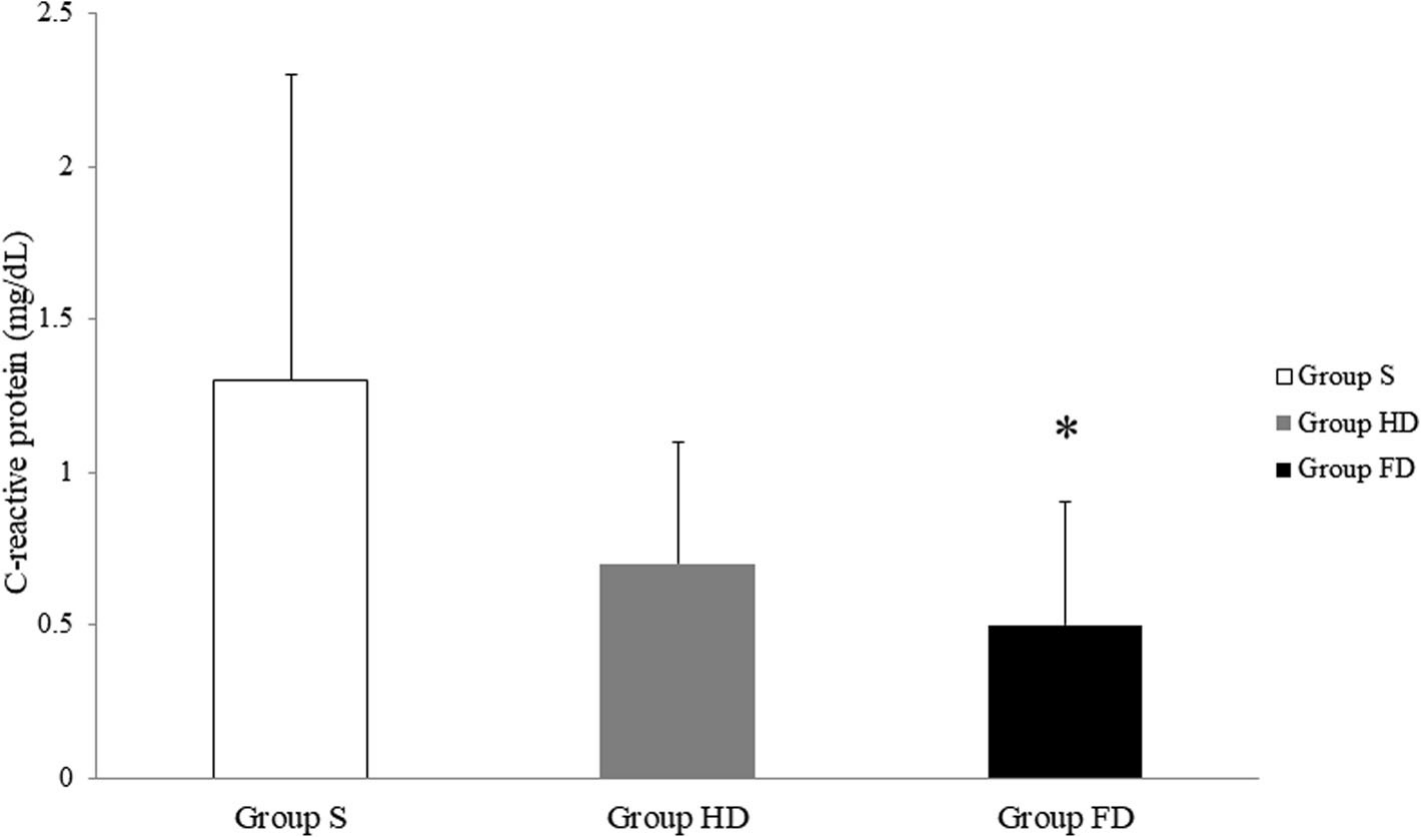
Comparison of C-reactive protein levels on postoperative day 1. Box shows the mean and error bar indicates SD. S, saline; HD, half dose; FD, full dose

The CRP concentrations on the next day following surgery were lower in the group HD and the group FD compared with the group S. The effects were dose-dependent, and the difference between the group S and the group FD was statistically significant (*P* = 0.037) (Fig. 2).

## Discussion

The major finding of this study is that the administration of dexmedetomidine significantly increased IFN-gamma/IL-4 and IL-17/IL-10 ratios in a dose-dependent manner in patients under surgical and anaesthetic stress. These findings indicate that dexmedetomidine, an alpha-2 agonist, probably shift the Th1/Th2 and T17/Treg balance toward Th1 and Th17, respectively, and thus plays an immunoregulatory role.

Initial T helper cells produce various kinds of cytokines. Depending on the signals received at stress state, they produce a more specific kind of cytokine [8]. On this account, T helper cells are subdivided by what kind of cytokines they secrete. Th1 cells produce IFN-gamma, by which macrophages and cytotoxic T lymphocytes are activated. Importantly, Th1 cells promote cell-mediated immune responses which may play a protective role and lead to fewer postoperative infection. On the other hand, Th2 cells, which produce IL-4, IL-5, and IL-10, driving B cells to produce immunoglobulins. As a result, Th2 cells promote humoral or antibody mediated immunity that leads to the suppression of cell-mediated immune responses [6, 8]. Therefore, shifting to either Th1 or Th2 cells can significantly influence subsequent state and direction of immune responses. Considering that surgery itself decreases the Th1/Th2 ratio [7], the infusion of dexmedetomidine during surgery might have had a beneficial effect on immunomodulation.

It has also been recently reported that there is an IL-23-dependent Th cell population which produces IL-17 [8–10]. This suggests a distinct third arm of T-cell effector repertoire: Th1, Th2 and Th17. IL-17 and IL-10, as cytokines of Th17 and Treg, respectively, and their ratio (IL-17/IL-10) were reported to play important roles in immunoregulation as the balance between inflammatory and immune regulatory cytokine is critical [9, 10, 12]. Our results showed that dexmedetomidine was associated with a decrease in IL-4 and IL-10, which are cytokines of Treg cells, and a skewing of the T17/Treg balance toward T17, as well as shifting Th1/Th2 balance toward Th1. These evidences suggest that any inflammatory response can be modulated by dexmedetomidine administration in a dose-dependent manner in patients who are stressed and have immune-compromised reactions due to surgery and anaesthesia.

The exact mechanisms by which alpha-2 agonist increases Th1/Th2 and Th17/Treg cytokine ratio are unclear. One possible explanation is based on that catecholamines and glucocorticoids secreted from the adrenal gland act on their receptors on T cells and macrophages, inhibit the production of Th1 cytokines and promote the production of Th2 cytokines, which results in decrease in Th1 to Th2 cytokines ratio [13]. It is thought that excessive activation of the sympathetic nervous system and the resulting immune-compromised response caused by immune system interactions during and after surgery were modified by central sympatholytic effects of dexmedetomidine. Another explanation is that, as was found in the animal study, macrophages have alpha-2 adrenoceptor on their surfaces [14], and clonidine, an alpha-2 agonist, binds to its receptor on macrophage and stimulate the secretion of IL-12, a potent inducer of Th1 cells, from mouse macrophage [15]. Dexmedetomidine, another alpha-2 agonist, might have acted as a similar immune modulator in patients with surgical and anaesthetic stress.

There have been a few studies on the impacts of anaesthetic agents or techniques on Th1/Th2 ratio. For patients undergoing neurosurgery, isoflurane was associated with decrease in Th1/Th2 ratio, on the other hand, propofol infusion prevented the ratio from decreasing perioperatively [16]. Whereas general anaesthesia decreased Th1/Th2 ratio, regional anaesthesia increased the Th1/Th2 ratio for patients receiving prostate surgery [17]. These studies focused on the changes in Th1/Th2 ratio depending on which anaesthetic agents or techniques were adopted. Through an experiment administrating dexmedetomidine to mouse twice a day for a week, Inada et al. [18] found that it leads to reducing Th1/Th2 ratio and triggers a shift to Th2, which shows conflicting data from our results. This diversion can be attributed to the fact that they administrated dexmedetomidine for a long time, and the sensitivity of alpha-2 adrenoreceptors might have been downregulated, which as a result drive decreased Th1/Th2 cell ratio [19, 20].

We found that half and full dose groups showed lower postoperative CRP concentrations compared to the saline group in a dose-dependent pattern. Increased concentration of CRP in the saline group may be due to stress responses to surgical stimulation, since pro-inflammatory cytokines, which is secreted as a stress response, stimulate hepatic production of CRP, an inflammatory marker [21]. Likewise, lower concentrations of CRP for patients in dexmedetomidine-treated groups may be a result of the decrease in stress responses by immunomodulatory role of dexmedetomidine.

The following points should be considered in interpreting the results of this study. First, we used whole blood sample to quantitate INF-gamma, IL-4, IL-17, and IL-10. Measuring plasma cytokines concentrations to estimate Th cell balance may be inaccurate because all IFN-gamma and IL-4 present in plasma are not derived solely from Th1 and Th2 cells. Although we did not perform flow cytometry to measure intracellular cytokines to accurately estimate Th cell balance, several other studies have been reported to describe the Th cell balance based on the analysis of whole blood sample by ELISA [22, 23]. In particular, Silva et al. [24] asserted that whole blood analysis and mono-nuclear cells techniques using peripheral blood samples are suitable for the study of cytokines production. Second, it relates to the clinical effect of shifting of the ratios of Th1 to Th2 and Th17 to Treg cytokines. We could not identify the relationship between the shifting of cytokine ratios and clinical outcomes, such as wound infection, or length of stay in hospital. It reflects the difficulty in comprehending whether the shifting of cytokine ratios by dexmedetomidine is an advantage or disadvantage. We attribute this into the fact that we included healthy patients undergoing minor surgery like laparoscopic cholecystectomy. However, several previous studies support overcoming the limitations of our study. Tan et al. [25] found that probiotics could increase the Th1/Th2 (IFN-gamma/IL-4) ratio to shift towards Th1 in traumatic brain-injured patients with decreased Th1/Th2 ratio, which led to decreased infection rates. The results of Cardinale et al. [7] were consistent with our study, where surgery itself decreased the Th1/Th2 ratio and the decreased ratio led to suppressed cell-mediated immunity in postoperative period. It has also known that Th1 and Th17 cells could be the major agents in inflammation in patients with reactive arthritis [9], and the imbalance of Th17/Treg plays important role in the pathogenesis of idiopathic thrombocytopenic purpura [10]. Thus, surgeons and anaesthesiologists should not ignore the influence of immunomodulation during surgical procedures on the perioperative outcomes of immunity. Future studies should include major surgical patients and focus on the clinical outcomes in terms of perioperative mortality and morbidity.

## Conclusions

We investigated the effects of dexmedetomidine, an alpha-2 adrenoceptor agonist, on the perioperative ratio of Th1 to Th2 and Th17 to Treg cytokines and postoperative level of CRP in patients undergoing laparoscopic cholecystectomy. The results indicated that the concentrations of IFN-gamma, a Th1 cytokine, and the ratios of IFN-gamma to IL-4 for Th1/Th2 cytokine ratios, were higher in the dexmedetomidine-treated groups with dose-dependent pattern than those in the saline group during and after surgery. The concentrations of IL-17, a Th17 cytokine, and the ratios of IL-17 to IL-10 for Th17/Treg cytokine ratios showed similar pattern. In addition, treatment with dexmedetomidine impeded the increase of CRP levels after surgery in dose-dependent manner. All in all, these imply that the use of alpha-2 agonist may modulate the immune response during and after laparoscopic surgery. Therefore, findings from the present study may provide significant information that contributes to developing strategies to regulate inflammatory response after surgery.

## Abbreviations

ANOVA: Analysis of variance
CRP: C-reactive protein
ELISA: Enzyme-linked immunosorbent assay
IFN-gamma: Interferon-gamma
IL-10: Interleukin-10
IL-17: Interleukin-17
IL-4: Interleukin-4
SD: Standard deviation
Th1: T helper 1
Th17: T helper 17
Th2: T helper 2
Treg: regulatory T cells
